# The Hystero Neurosis of the Stomach in Pregnancy

**Published:** 1881-03

**Authors:** John S. Warren

**Affiliations:** New York. Physician to Department of Diseases of Women, Demilt Dispensary


### Selections.

### THE HYSTERO NEUROSIS OF THE STOMACH IN PREGNANCY.

By JOHN S. WARREN, M.D., New York.
Physician to Department of Diseases of Women, Demilt Dispensary.

The tendency of modern therapeutics is to constantly seek specifics for diseased conditions.

One of the results of this search is the too frequent announcement of such specifics founded upon insufficient experience, and hence followed by disappointment.

The reason that this disposition exists, is not only on account of our increased knowledge derived from pathological study, whereby we learn the etiology of disease; upon the development of chemical science, and the consequent production of new remedial agents; upon the physiological investigations as to the action of medicines; but especially because some of our most valuable remedies have come to us empirically.

The subject which I have selected for your consideration, "The Hystero-Neurosis of the Stomach in Pregnancy," is common-place, and it may be, uninteresting, has appeared to me to especially illustrate the folly of this continual search for specific medication.

The whole range of remedies has again and again been reviewed for this purpose, and scarcely a week or month passes without hearing that the discovery has been made, only to be followed by inevitable inefficiency and merited neglect.

The vomiting of pregnancy has not heretofore received the consideration from the friends and physicians that it deserves. Too long the custom has been with the former, to make the pregnant woman believe that in her condition all things are possible. And with the latter, that unless the trial of a few routine medicines relieved her distress, she must patiently await the period of quickening for the abatement of her sufferings.

Too long has this aggravating and often dangerous disease, both to the mother and child in utero, been treated upon the supposition that an engorged uterus was its sole and only cause; and not as a disturbance capable of being produced, or at least greatly increased, by the functional or organic derangement of other organs.

A pretty large experience with women suffering from this complaint, during the past five years, in the Female Department of Demilt Dispensary, and including several hundred patients, as well as in private practice, has led me to believe that much of the ill success that has attended the treatment of this disease, has been due often to the careless and empirical way in which it has been managed; and without attempting, in each individual case, to make a correct diagnosis of the cause of the stomach irritation, or to distinguish between the vomiting *of* pregnancy and the vomiting *in* pregnancy.

Trousseau says (vol. iii., p. 18) "that to cure, and, when that cannot be done, to alleviate the sufferings of patients, is the object of medicine. And while treatment is dependent upon the experience, talent and tact of the physician, it is still more subordinate to the nature of the disease which he wishes to cure, to particular conditions under which the disease exists, to the peculiarities of the organization of the patient, a knowledge of the symptoms, and a proper understanding of their causes and natural history."

Without further premise I will say, that in the management of the vomiting of pregnancy, I believe it is absolutely essential to ascertain at the onset, if possible, whether the symptom is due purely to the sympathetic disturbance proceeding from a congested uterus, or is it dependent upon other causes and disorders of other organs, each capable of producing the same symptoms at other times than during pregnancy? and to which, if our early attention is directed, and the proper treatment applied, the gastric trouble will be much lessened, if not entirely abated.

Among the first of these, and not the least important, is the effect which the emotional or hysterical element may produce at this time. In some people, if not in all, emotion, in some form or other, connects itself with every thought, word or action, giving at one time zest to existence, at another depressing even the simplest of our powers. The stomach is known to be most intimately connected with morbid emotions; so that the function of digestion may not only be arrested and vomiting occur, but even that symptom may happen when the stomach is empty, if distressing or unpleasant thoughts are present to excite.

Dr. Greenhalgh (London *Lancet*, 1868,) says "that the form of vomiting, which but few pregnant women escape, occurring very early in pregnancy, the appetite being unimpaired, is characterized by the ejection in the morning of a little glairy mucus tinged with bile, in which there is no evidence of undigested food. This form occurs most frequently and severely in those who are of a nervous and hysterical temperament, and who have suffered before from some uterine ailment. It also more frequently affects the rich than the poor, owing probably to the more highly altered state of the nervous system and greater mental activity of the former."

In such cases it seems that vomiting is but a symptom of that morbid irritability which is so common in females; that depends upon the impressibility of their nervous organizations, and which is set wrong by any slight derangement of their general health. Cannot the vomiting in the sympathetic husband at this time be attributed to emotional causes?

Again may be mentioned, as powerful agents in exciting this trouble, all those conditions that help to increase the natural uterine engorgement, whether it is the uterus, prolaps d and crowded into the bony pelvis by reason of previous subinvolution, a sharp ante or retroflexion, a lacerated cervix or eroded os uteri, an undue tension of the muscular fibres of the neck, fibroid tumors, or an obstinate constipation. Any of these conditions are capable in women of not only seriously disordering the digestive function at any other time, but even of producing the symptom of nausea and vomiting, and may, in many cases, if complicating the reflex irritation following conception, both greatly increase its severity and at times render it intractable.

Corroborative of this, many cases and reliable authorities might be quoted, if necessary.

Even Bennet asserted in his ardent writing upon uterine inflammation, that in his so-called "ulceration" existed the *keystone* of the diseases of the pregnant state, especially those cases of obstinate vomiting which sometimes defied all medicinal treatment, and advised the local application of the solid nitrate of silver for a certain cure. But a more eminent authority, is Dr. Marion Sims, who not long since detailed (London *Lancet*) several extreme and hopeless cases of pregnant vomiting which yielded at last to the same application applied to an eroded os.

Dr. Graily Hewitt too soon decided that *all* cases of this disease had for their cause a forward flexion of the uterus. Still, we all know that this displacement is not infrequently a complication which, if relieved, gives the stomach rest and enables digestion and nutrition to go on.

At times, too, a slight expansion of the servical canal by Copeman's method has proved to be the only and a very positive remedy for arresting the distress.

Again, I will refer to the influence exerted by the derangement of other organs of the body in inducing this disease, whether existing prior to or occurring with the commencement of gestation, and for this purpose I can do no better than to briefly refer to a remarkably terse and suggestive paper read by Dr. Robert Barnes, of London, before the American Gynæcological Society in 1876, and published in the first volume of "Transactions," wherein he speaks of the many and important changes which happen in the female economy during the process of gestation, notably referring to the extraordinary activity of the lymphatics, manifested by the rapid wasting of fat and the marked changes that take place in the glandular system, especially in the thyroid, spleen, liver and kidneys. What exact importance the disorder of the first two mentioned may possibly be to the disease in question we are not prepared to positively assert. But the influence of the two last-named—the liver and kidneys—whether their disorder is functional or organic, alone or complicated with other conditions, are too important to be overlooked; and all eminent gynæcologists of the present day are ever ready to emphasize the necessity in the treatment of all uterine disease of keeping them in their proper functional capacity.

In the latter months of pregnancy albuminuria may sometimes exist without the usual signs of its presence, by dropsy, headache, disordered vision, etc., and may be the cause of vomiting, as cases on record will attest. Here, too, the friends and physician even, may be disposed to attribute it to a sympathetic disturbance, especially if the sickness has occurred in the earlier months and has once ceased. So insidious may be its progress, that it is not until dangerous or perhaps fatal symptoms appear that the true disease is manifest. In this period of gestation the possibility of the existence of this form of nephritis without the commoner manifestations should not be lost sight of, but frequent and repeated urinary examinations should be made.

Without a further detail of causes which may contribute to or directly produce this symptom, even when at first it may appear to be reflex, I will only briefly relate two cases which have recently occurred to me in private practice, and while I have been preparing this paper.

The first, a young married lady, who had borne one child several years ago, and who had since been perfectly regular, came to me, fearful that she might again be pregnant. She had passed nearly two months without being unwell. Although I had known her for many years to be in good health, she at this time was anæmic and debilitated. Her symptoms were those of general prostration, with nausea, and vomiting of food during the day.

A first, thinking that the latter symptom was especially significant of pregnancy, I asked about her previous experience, and she informed me that she had suffered considerably from vomiting with her first child, but she remarked: "I always become sick at the stomach when I am fatigued or do any laborious work." Doubtful as to her condition, but simply putting her upon tonic treatment, her menstrual period again appeared naturally, and she has since continued to improve in health.

Another case was in a lady of high respectability and good connections, who had been several times pregnant, and who after six weeks' suppression, became violently sick and vomited incessantly.

At this time she informed me that a small quantity of brandy was the only thing that her stomach would retain. This I directed her to stop using, and after ordering the bromide of sodium in full doses, to be followed by a remedy which I shall later mention, I left her until the next day, when I found her condition greatly improved. In a few days, being again summoned, she told me her vomiting was as bad as ever. Again interdicting the use of brandy, which I susp cted she was in the habit of taking, and which suspicion was confirmed by the advice of a lady friend, her complaint ceased, although afterward she succeeded, through effo ts on her part, in producing an abortion.

These two cases illustrate the last and only cause of this neurosis which I shall enumerate; the former showing the influence of physical effort in the pr duction of stomach disorder, the latter exhibiting the effect of over-stimulation by the use of alcohol, in bringing about the same results; the one common, the other less frequent, but highly important and exceedingly difficult to deal with, particularly if the patient be a gentlewoman, for she will never acknowledge her weakness until recurrent attacts render it patent, as women are much more secretive in the indulgence of the habit than men.

And now, if sufficient evidence has been offered to show that this disease has many and varied influences, aside from pregnancy, capable of producing or complicating it, does it not deserve the same attention, when demanding treatment, as other diseases or symptoms? I claim that it does, and that its successful management must always be dependent upon the correct diagnosis of its cause, whether simply reflex in character or due to many other conditions or disorders, a few of which I have mentioned.

The treatment, then, of the vomiting of pregnancy resolves itself into the correction of all disturbances, functional or organic, as far as possible, which are known to excite dyspeptic symptoms, before a simple irritation becomes a confirmed gastritis, and the stomach refuses to receive remedies most appropriate to relieve the original trouble. Among these, oftener than others, the emotional element and a constipated habit, with its attendant flatulence and discomforts, accompany the pregnant state, and should receive early and prompt attention. For the relief of the former, no remedies at this time equal in efficiency the bromides of sodium and potassium exhibited in full doses. And here it may not be amiss to state, that in order to secure their full effect these medicines must be administered at the proper time, generally late in the day or at bedtime, and when the stomach is empty.

The constipation can be overcome by any simple laxative, as the comp. liquorice powder, or any other harmless medicine or formula, or if obstinate, copious and repeated enemata of tepid water will unload the rectum of the hardened faces or scybala which so frequently occur in women.

Finally, when all other causes are excluded, the constipation relieved, and the emotional element controlled, and we come to consider the purely sympathetic disorder following conception; in short, when we have to deal with the uncommon vomiting, due simply and solely, so far as we can see, to the impregnation of a healthy uterus in a healthy woman, I have found many of the remedies which have been called specifics to sometimes relieve, but often to fail. But the one remedy which, in my hands, has before all others proved the most efficient for alleviating the distress, if not for curing the complaint, is Fowler's solution of arsenic, administered in drop doses upon an empty stomach. When thus given, and with a restricted diet, it has seemed to me to come nearer to a specific for this neurosis than any other. Indeed the effect is at times almost magical, and when continued for a considerable period, and given in larger doses when the stomach contains food, affords, in my opinion, a nerve tonic highly essential to women in the pregnant state, and which no other remedy can equal.

Frequently, however, after its continuance for a considerable time, benefit comes from suspending its use and substituting the nitro-muriatic acid with tinct. nux vomica, particularly if there be any inactivity of the liver or kidneys, or anorexia exists.

In conclusion, every pregnant woman, and especially those suffering from vomiting, should be placed under the best possible hygienic conditions, the diet carefully regulated, sufficient exercise enjoined, and above all the mind should be actively employed.—*Medical Record.*

